# Supplementary figures and images for: Interactions of semiconductor Cd-based quantum dots and Cd2+ with gut bacteria isolated from wild Salmo trutta fry
Source: PeerJ. 2022 Sep 15;10:e14025. doi: 10.7717/peerj.14025 (PMC9482770; doi:10.7717/peerj.14025)

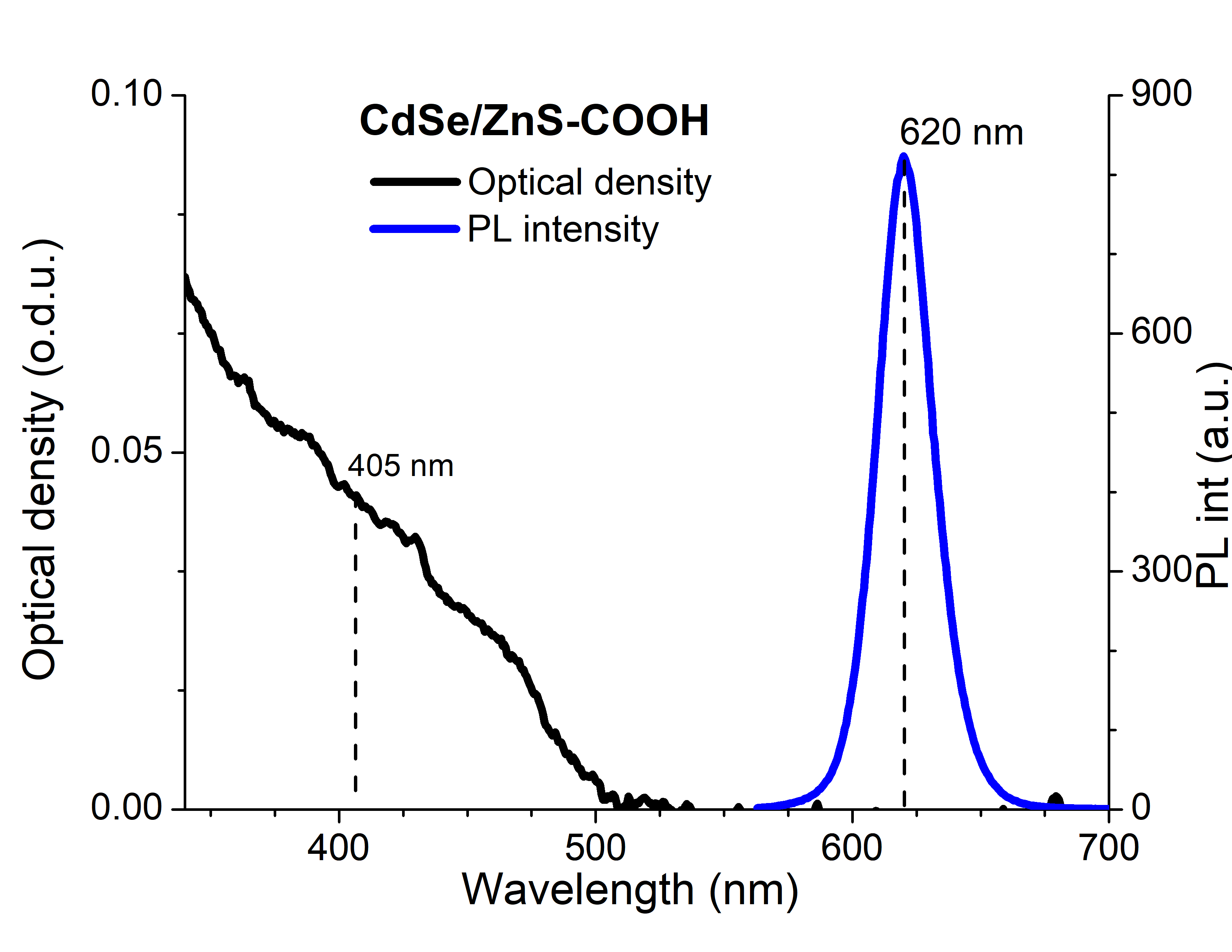

Supplement: Supplemental Information 1 — An excitation wavelength for PL was set at 405 nm, the excitation slit was 10.0 nm and the emission slit was 2.5 nm. [file peerj-10-14025-s001.png]

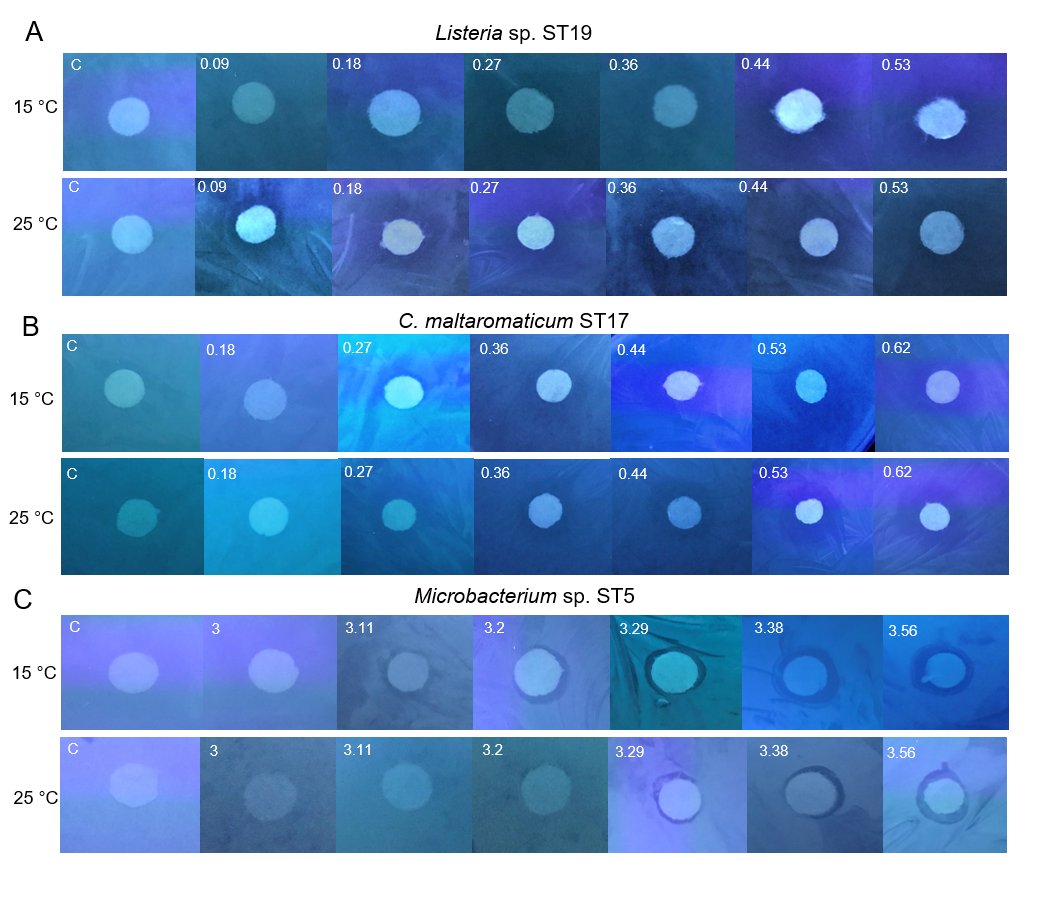

Supplement: Supplemental Information 3 — Antibacterial efficacy on Gram-positive bacteria: Listeria sp. ST19 (A), C. maltaromaticum ST17 (B), Microbacterium sp. ST5 (C) at two tested temperatures (15˚C and 25 ˚C) at pH 7.0. [file peerj-10-14025-s003.png]

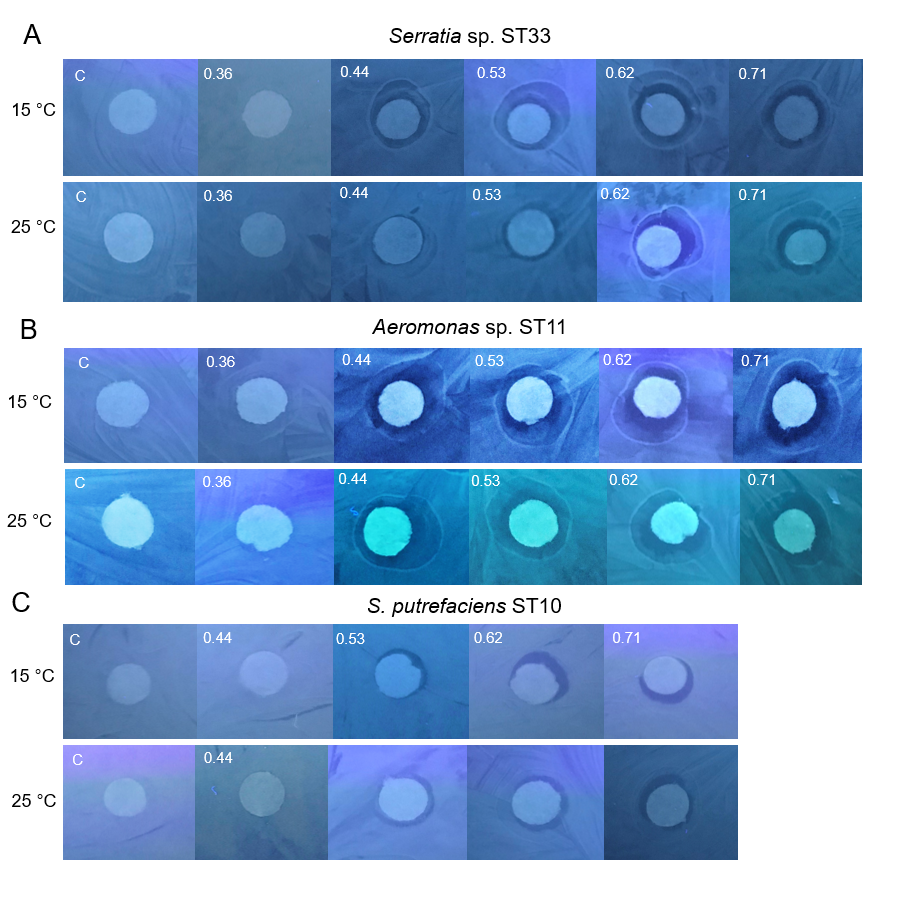

Supplement: Supplemental Information 4 — Antibacterial efficacy on Serratia sp. ST33 (A), Aeromonas sp. ST11 (B), and S. putrefaciens ST10 (C) at two tested temperatures (15˚C and 25 ˚C) at pH 7.0. [file peerj-10-14025-s004.png]
