# Supplementary material for: Interactions of semiconductor Cd-based quantum dots and Cd2+ with gut bacteria isolated from wild Salmo trutta fry
Source: PeerJ. 2022 Sep 15;10:e14025. doi: 10.7717/peerj.14025 (PMC9482770; doi:10.7717/peerj.14025)
Supplement: Supplemental Information 2 [file peerj-10-14025-s002.pdf]

Supplementary Table S1: Minimum inhibitory concentrations (MIC) of Cd^{2+} against isolated *Salmo trutta* fry gut bacteria at pH 7.0.

Genus/species	Isolates No.	Sequences similarity (%)	MIC of Cd^{2+}, mg/L (mM)			
			15 ºC	Zone of inhibition mm (mean ± SD)	25 ºC	Zone of inhibition mm (mean ± SD)
**Gram-positive bacteria:**						
Carnobacterium maltaromaticum	ST6	100	60 (0.53)	9.7 ± 0.5	50 (0.44)	10.1 ± 0.6
C. maltaromaticum	ST17	100	30 (0.27)	7.7 ± 0.5	30 (0.27)	7.6 ± 0.2
***Listeria* sp.**	ST19	99	20 (0.18)	8.6 ± 0.2	10 (0.09)	8.6 ± 0.2
***Microbacterium* sp.**	ST5	99	350 (3.11)	8.1 ± 0.2*	370 (3.29)	7.4 ± 0.2
**Gram-negative bacteria:**						
***Aeromonas* sp.**	AL8	100	80 (0.71)	7.7 ± 0.5*	80 (0.71)	6.7 ± 0.5
***Aeromonas* sp.**	ST11	99	50 (0.44)	7.2 ± 0.4	50 (0.44)	7.5 ± 0.6
***Aeromonas* sp.**	ST15	99	50 (0.44)	6.9 ± 0.1	50 (0.44)	7.0 ± 0.2
A. popoffii	AL2	99	60 (0.53)	7.7 ± 0.5	60 (0.53)	7.3 ± 0.4
A. salmonicida	AL13	99	70 (0.62)	6.7 ± 0.5	70 (0.62)	7.4 ± 0.5
A. salmonicida	AL20	99	80 (0.71)	6.2 ± 0.1	80 (0.71)	6.7 ± 0.5
A. salmonicida	ST8	99	70 (0.62)	7.7 ± 0.5	80 (0.71)	7.7 ± 0.5
A. salmonicida	ST9	99	70 (0.62)	8.0 ± 0.1*	70 (0.62)	7.6 ± 0.1
A. salmonicida	ST16	99	60 (0.53)	7.9 ± 0.2*	60 (0.53)	7.5 ± 0.3
A. sobria	AL10	99	70 (0.62)	7.3 ± 0.4	70 (0.62)	7.5 ± 0.2
A. sobria	ST3	99	60 (0.53)	7.2 ± 0.0*	60 (0.53)	8.6 ± 0.2
A. sobria	ST4	99	60 (0.53)	8.4 ± 0.5	60 (0.53)	7.7 ± 0.5
A. sobria	ST7	99	70 (0.62)	7.2 ± 0.2*	70 (0.62)	8.1 ± 0.2
A. sobria	ST12	99	70 (0.62)	7.0 ± 0.3	70 (0.62)	7.1 ± 0.2
A. sobria	ST18	99	60 (0.53)	7.7 ± 0.5*	70 (0.62)	6.7 ± 0.5
***Buttiauxella* sp.**	ST32	99	70 (0.62)	7.1 ± 0.1*	70 (0.62)	6.7 ± 0.1
***Serratia* sp.**	ST33	98	50 (0.44)	7.5 ± 0.4	60 (0.53)	7.3 ± 0.5
Shewanella putrefaciens	AL18	99	80 (0.71)	6.7 ± 0.4*	80 (0.71)	7.4 ± 0.4
S. putrefaciens	ST1	99	60 (0.53)	7.5 ± 0.4	60 (0.53)	7.0 ± 0.3
S. putrefaciens	ST10	99	60 (0.53)	6.8 ± 0.2*	60 (0.53)	7.6 ± 0.2
S. putrefaciens	ST13	99	60 (0.53)	7.9 ± 0.2*	60 (0.53)	7.4 ± 0.2
S. putrefaciens	ST14	99	60 (0.53)	7.2 ± 0.3	70 (0.62)	7.6 ± 0.3

*Statistically significant differences of MIC inhibition zones mm, $p \leq 0.05$
